# Reciprocity in Interaction: A Window on the First Year of Life in Autism

**DOI:** 10.1155/2013/705895

**Published:** 2013-05-20

**Authors:** Fabio Apicella, Natasha Chericoni, Valeria Costanzo, Sara Baldini, Lucia Billeci, David Cohen, Filippo Muratori

**Affiliations:** ^1^Scientific Institute “Fondazione Stella Maris”, Viale del Tirreno, 331, I-56018, Calambrone, Pisa, Italy; ^2^Institute of Clinical Physiology, National Council of Research (CNR), Via G. Moruzzi 1, 56124 Pisa, Italy; ^3^Department of Child and Adolescent Psychiatry, Groupe Hospitalier Pitié-Salpétrière, APHP, Université Pierre et Marie Curie, 47 bd de l'Hôpital, 75013 Paris, France; ^4^Department of Developmental Neuropsychiatry, University of Pisa, Viale del Tirreno, 331, I-56018, Calambrone, Pisa, Italy

## Abstract

From early infancy onwards, young children appear motivated to engage reciprocally with others and share psychological states during dyadic interactions. Although poor reciprocity is one of the defining features of autism spectrum disorders (ASDs), few studies have focused on the direct assessment of real-life reciprocal behavior; consequently, our knowledge of the nature and the development of this core feature of autism is still limited. In this study, we describe the phenomenon of reciprocity in infant-caregiver interaction by analyzing family movies taken during the first year of life of 10 infants with ASD and 9 infants with typical development (TD). We analyzed reciprocal behaviors by means of a coding scheme developed for this purpose (caregiver-infant reciprocity scale (CIRS)). Infants with ASD displayed less motor activity during the first semester and subsequently fewer vocalizations, compared to TD infants. Caregivers of ASD infants showed in the second semester shorter periods of involvement and a reduction of affectionate touch. These results suggest that from the first months of life a nonsynchronic motor-vocal pattern may interfere in different ways with the development of reciprocity in the primary relationship between infants later diagnosed with ASD and their caregivers.

## 1. Introduction

Reciprocity can be defined as an interactive condition in which two individuals mutually respond to each other while performing activities together [1]. This attitude is present from the first moments of life, when an infant is naturally orientated towards the mother's face and responds preferentially to it. Reciprocity is necessary in order to reach shared goals and consists of symmetrical exchanges characterized by finely tuned turn-taking [2, 3]. From the first interactions with their caregivers, typically developing infants (TD) show a natural aptitude for engaging in joint action and for sharing psychological states [4–6]. Mutual exchanges exist from the beginning when each member of the dyad is responsive to the other, trying to engage the other in turn and changing his/her behavior according to the other's solicitations [7]. Poor reciprocity is one of the defining features of autism spectrum disorders (ASDs), a class of neurodevelopmental disorders that disrupt regular interactions in the social realm [8]. A widely shared hypothesis is that abnormalities in the processing of and in responding to stimuli coming from the social environment are in action from the first stages of life, with cascading effects on mutual exchanges in dyadic interactions. Despite the fact that a reduced reciprocity is frequently reported as one of the earliest signs of autism, few studies have directly assessed reciprocity during infant-parent interactions in a naturalistic setting; consequently, the nature and the developmental course of this core feature in autism are still open issues.

Some studies on early autism, both retrospective and prospective, have described behavioral difficulties that are in some way linked with the concept of reciprocity. Palomo et al. [9] suggested that in infants with ASD, compared to TD infants, there are some differences in the ability to interact reciprocally, because of a reduction in the capacity of the former to engage with the other and to orient toward social stimuli. Wan et al. [10] found that infants at-risk of ASD showed lower levels of liveliness, that is, a general tendency toward less activity during exchanges. We have described a general difficulty in maintaining social engagement and in accepting invitation [11]. Other studies [12, 13] have revealed that during mutual exchange between infant and parent, while the former serves as the leading member in a typical dyad, this does not happen in dyads with an infant with ASD. Infants with these social difficulties offer their parents,who are in need of both responses and prompts from their infants, fewer opportunities for interaction. While infants' social difficulties have been sufficiently analyzed and assessed, their effects on parental attitude have received less attention. Some studies have described longitudinal changes in the intensity and quality of parental solicitations. Saint-Georges et al. [14] focused on sequentially analyzed interactions, and they found that parents of infants with ASD (1) respond to their infants at the same level as parents of infants with TD but (2) show a more directive interactive style characterized by an increase in stimulating and touching activity. Congruently, Doussard-Roosevelt et al. [15] and Trevarthen and Daniel [16] found that parents of children with ASD overstimulate their infants as a result of their inactivity. Van Ijzendoorn et al. [17] observed that parents of children with ASD tended to answer their child's signals with more physical contact and fewer verbal approaches. We have described [11] a reduction in caregiver behaviors aimed at downregulating the infant's arousal and mood as one of the first expressions of lack of initiative in infants with ASD.

This evidence of an early alteration in parent-infant interaction suggests that the first signs of ASD could be better understood by observing interactive dynamics rather than the increase or decrease in single behaviors of one member of the dyad. As stated by Thomas and Martin [18] and by Anderson et al. [1], parent-infant reciprocity is better studied by analyzing the effects on the other member of each dyad member's behavior.

On the basis of these assumptions, we have hypothesized that reciprocity can be better understood by analyzing dyadic behaviors that occur in simple caregiver-infant interactions observed in family home videos, a valid method for observing ecological interactions from the first stages of life. To describe interactions specifically in terms of reciprocity, we have developed the caregiver-infant reciprocity scale (CIRS) which consists of items referring to mutual exchanges (such as involvement or responsiveness) and single or molecular behaviors (such as vocalization for infants or name prompt for caregivers). The CIRS was developed in order to describe and evaluate how infants try to involve and to respond to the other's bids; according to the literature [9, 11], we expected, in infants with ASD, a reduced frequency of behaviors used to involve and to maintain the other in interaction. At the same time, the CIRS enabled us to explore the effect of the infant's behavior on parents' interactive attitudes; according to the literature, we expected some differences in terms of type and quantity of stimulations but not in terms of global availability [17]. The CIRS was also developed to explore whether and to what extent abnormalities in the frequency of motor behaviors contribute to disturbing the development of the atypical infant's ability to engage in interactions: our hypothesis, based on a previous exploratory study, was that abnormalities in motor activity can constitute a warning signal for difficulties in reciprocity.

## 2. Methods

### 2.1. Participants

We studied retrospective home movies from the first year of life of two groups of children. The first group was composed of 10 children (M/F: 9/1) with a diagnosis of autism spectrum disorder (ASD) performed by a child psychiatrist according to the DSM-IV-TR criteria [19] and confirmed by ADOS-G and ADI-R scores. At the time of the diagnosis, their ages ranged between 4 and 6 years. They were also administered Griffiths mental developmental scale-ER in order to determine intellectual functioning (mean IQ: 59.26; sd: 8.49). The second group was composed of 9 typically developing (TD) children who were recruited among children attending a local kindergarten; these children did not present any abnormal medical or developmental condition as confirmed by nonclinical scores using the child behavior checklist [20]. At the time of recruitment, the TD children were aged between 4 and 5 years. The videos used in the study were taken from a larger collection of videos held at the Stella Maris Institute, University of Pisa, Italy.

### 2.2. Video Collection and Editing Procedures

Families were asked to provide any videotape recorded during their child's first year of life. Copies of the videos were made and coded with an ID number to preserve confidentiality. Written informed consent was collected from participating families. The selection of the video clips was carried out by a research assistant *naive* to the children's diagnoses. Only sequences where the child was involved in an interaction with a visible caregiver were selected for the study. Sequences were selected in terms of situation type. We included face-to-face interactions in various circumstances, such as bath time, nutrition time, and play time. We also included face-to-face interactions *per se* (i.e., the baby on the changing table and the mother in front of him/her talking in motherese). Birthday parties and sequences with several adults and/or children simultaneously present in the video were excluded.

A *t*-test was performed to check that the selected material was comparable across groups in terms of total duration and number and type of situations. In accordance with other studies in the literature [11, 21, 22] which analyzed the developmental pattern of infants across the first year of life, we considered the first and second six months of life separately, in order to take into account the multiple changes which take place during this period. The total duration of the videos analyzed was 46 minutes for the age period 0–6 months (hereinafter T1), and 51 minutes for the age period 6–12 months (hereinafter T2). We analyzed a total of 81 interactive sequences for infants with ASD and 80 for TD infants. 

### 2.3. Measures

We developed the caregiver-infant reciprocity scale (CIRS) on the basis of previous research using the infant caregiver behavior scale (ICBS) developed by Muratori et al. [11] and the emotional availability scales (EASs) by Biringen [23]. We were interested in studying caregiver-infant interaction in terms of reciprocal involvement (i.e., the attempt to attract the other into interaction) and responsiveness (i.e., the behavioral response to the other's attempt to start an interaction), focusing on the specific behaviors which characterize these items rather than describing these capacities in terms of level (as does the EAS). The CIRS (see Table 1 for the description of the items) is composed of 12 items describing both caregiver and infant behaviors used to respond to or involve the other (6 items refer to the caregiver, and 6 items refer to the infant). For both sets of items, 2 (involvement and responsiveness) are evaluated as states, in that they represent the length of time spent on either activating or responsive behaviors. Next to these items, 4 other items, referred to as events, are used to describe more specifically the single behaviors that are enacted to elicit a response from the other member of the dyad or to respond to the other's solicitations. For these latter items, only the frequency and not the duration of each is recorded. For the purpose of our study, we chose to code only those behaviors which were directed to the other member of the dyad and specified for each function in terms of involvement or responsiveness. In this way, vocalizations of the caregiver directed to another adult, or behaviors of the infant which were not related to the interaction with the caregiver, were not taken into account.

### 2.4. Computer-Based Coding System

The Observer XT 10.0′ [24], a professional software for the collection, management, and analysis of observational data, was used. This software had already been used to study behavioral patterns in infants with autism and its feasibility had been demonstrated [11]. The Observer was configured for the application of the CIRS to our videotapes. We have used the “Observer Video-Pro,” a software from the Observer package, which is able to read time information directly from the video, allowing an accurate event timing at the chosen playback speed.

### 2.5. Coders' Training and Intercoder Agreement Procedures

Two coders (NC and VC), with an undergraduate degree in psychology, were trained to use the coding system, by observing and coding videos of both TD infants and infants with ASD, who were not included in the study. The two-month training period enabled the coders to become familiar with the meaning of the items, to identify them correctly, and to acquire ability in coding procedures. In addition, the coders were required to achieve a satisfactory level of agreement between them (Cohen's Kappa ≥ 0.70) and with an expert clinician (FA) in two matched sequences for each age range. The tolerance window regarding time discrepancy between coding was set at 1 s for event behaviors and at 3 s for state behaviors. The coding recorded outside these windows was reported as a coding error and was considered a disagreement. In order to avoid any interpretation biases, the two coders were blind to group membership. Intercoders agreement was calculated for each item, and the values of *k* were ≥ 0,70. The mean Intercoder reliability, calculated directly by the Observer XT, showed satisfactory agreement (*k* = 0.81). In order to verify the ongoing agreement, 25% of the collection of sequences were coded by both coders. The mean Intercoder agreement calculated on these sequences was *k* = 0,84, and the values of *k* were ≥ 0,70. In all cases of discordance between coders, a third coder's (FA) advice was used.

### 2.6. Data Analysis

Due to the different length of video sequences of each child, the frequencies and the duration of each behavior were converted, respectively, to a ratio number of behaviors per time (hereinafter rate/min) and to a percentage duration (hereinafter % duration) calculated between the total duration of a behavior and the duration of the sequences available for the same subject [11, 21]. Two additional items were further created for the purpose of analysis, by merging activating behaviors—with motor activity and attuned behaviors—with motor activity in a unique item named total motor activity and by merging activating behaviors—with gaze and attuned behaviors—with gaze in a unique item named total gaze, in order to evaluate to what extent these behaviors are used, independently from their purpose (to involve/respond to the other person).

Rates and percentages of all CIRS items were compared using Student's *t*-test for independent samples in order to detect “between group” differences (separately for T1 and T2) and Student's *t*-test for paired samples to detect “within group” differences between age periods (separately for ASD and TD). Statistical analysis was performed using SPSS version 16.0 [25]. Level of significance was set at *P* < 0.05.

## 3. Results

Results are reported in Table 2, for infant behaviors, and in Table 3, for caregiver behaviors.

### 3.1. Group Comparison

Both at T1 and at T2 there were no significant differences between groups in the rate/min and in the percentage duration of the infant states (Table 2). At T1 infants with ASD showed a significantly lower result for the following events: activating behavior—total and total motor activity. At T2 infants with ASD showed a significantly lower result for the event vocalizations—total and in particular fewer vocalizations—of responsiveness.

As for caregiver behaviors (Table 3), both at T1 and at T2 no significant differences were found between groups either for states or events with the exception of the less affectionate touch—of responsiveness in caregivers of infants with ASD at T1.

### 3.2. Age Group Comparison

In TD infants (Table 2), a significant increase was found in the percentage duration of the state responsiveness. A significant increase was also found in the rate/min of activating behaviors—with motor activity, attuned Behaviors—with motor activity, and total motor activity. Vocalizations—total and vocalizations—of responsiveness showed a significant increase too. Age group comparison did not show any significant differences in TD caregivers either for states or events (Table 3).

In infants with ASD (Table 2), no significant differences were found for states in the transition between T1 and T2. A significant increase was found in activating behaviors—total, particularly in activating behaviors—with motor activity, and in total motor activity. In addition, a significant decrease was found in vocalizations—total. 

Caregivers of infants with ASD (Table 3) showed a significant decrease in the percentage duration of the state involvement and in the rate/min of affectionate touch, taking into consideration both affectionate touch—Total—and affectionate Touch—of involvement.

## 4. Discussion

**Figure 1:**
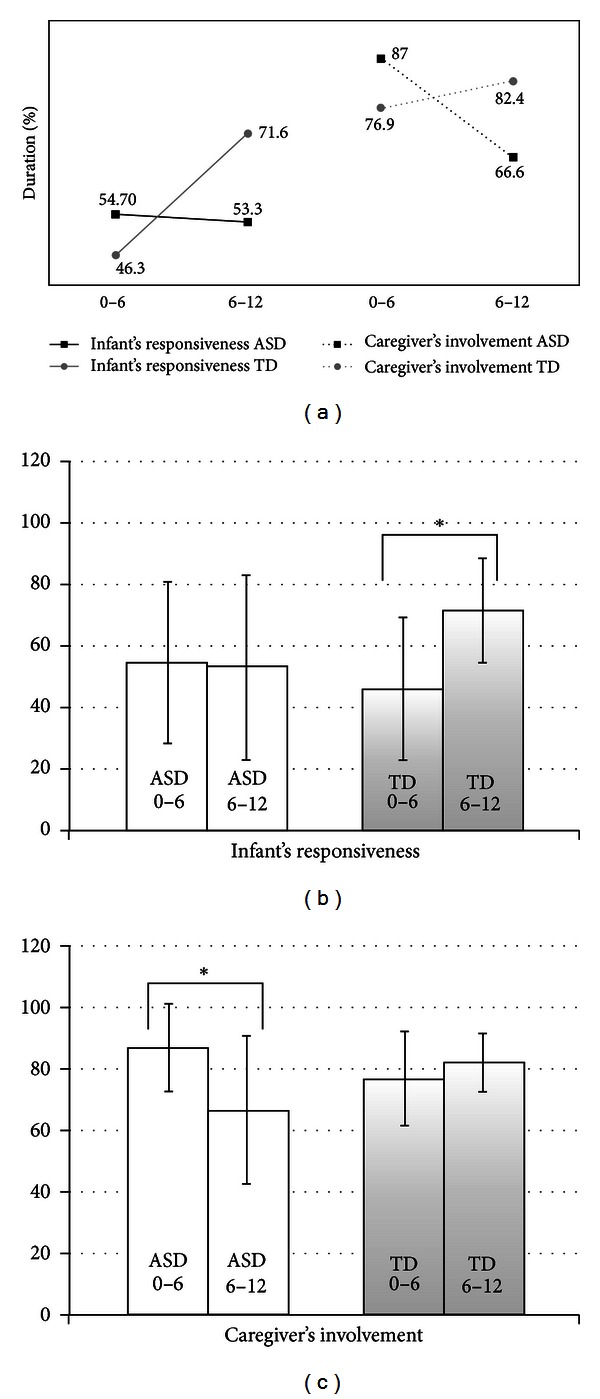
Trajectories of infant's responsiveness versus caregiver's involvement. (a) Trajectories (from T1 to T2) of infant's responsiveness (b) and caregiver's involvement (c). TD infants increase their responsiveness from T1 to T2 while in infants with ASD responsiveness remains stable. Caregivers of infants with ASD tend to decrease the time they spend in involvement. (b), (c) Means and standard deviations (error bars). **P ≤ 0.01; *P ≤ 0.05.

This study described both segmental and longitudinal peculiarities in the context of the early developing relationship between a small group of infants with ASD and their primary caregivers. Considering the small sample size, it provides a provisional in-depth model for understanding the onset and the perpetuation of deficits in social reciprocity. First of all, we found that while the time the dyads spent in involvement and responsiveness did not differ across groups, quite opposite trajectories emerged during the first year of life, suggesting that two different reciprocity profiles may be under construction in infants with ASD compared to TD infants (see Figure 1). In contrast with the TD profile in fact, the ASD profile is characterized by the absence of a growth in the infants' responsiveness and by the parallel decrease in the time their caregivers spend attracting their attention in order to engage them in an interaction. We could hypothesize that differences in the parents and infants' profiles are interconnected and that the parent's level of involvement may be modulated by the infant's responsiveness and *vice versa*.

**Figure 2:**
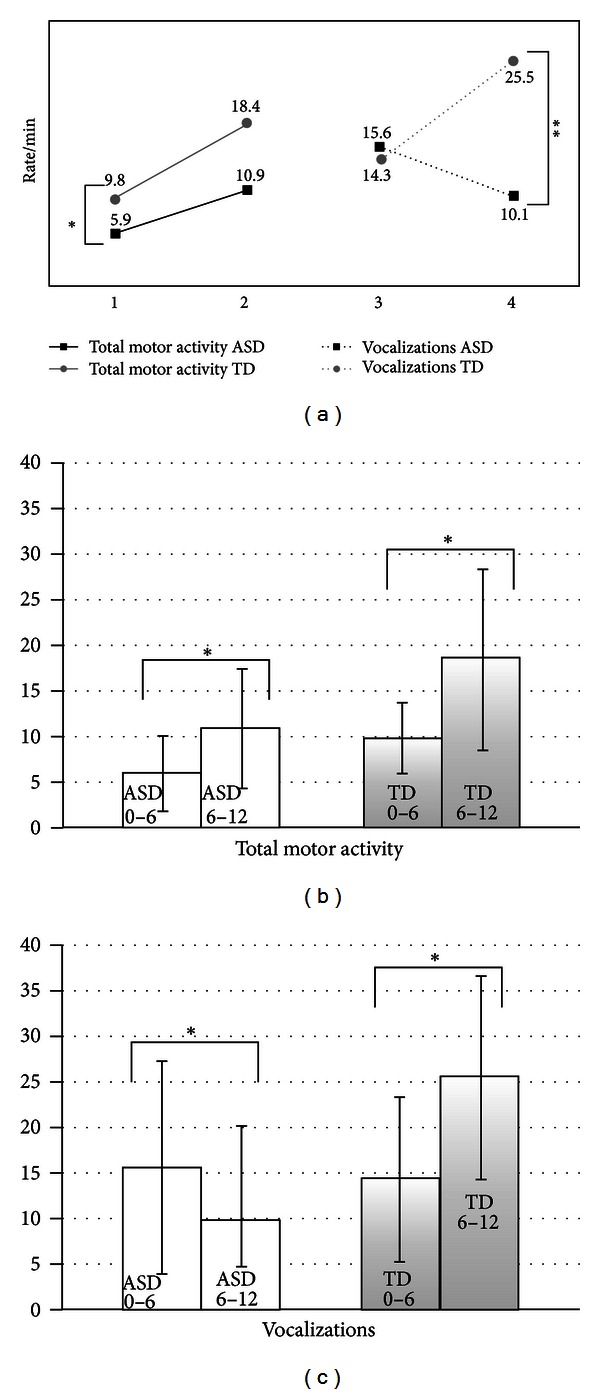
Trajectories of infant's total motor activity versus infant's vocalizations. (a) Trajectories (from T1 to T2) of total motor activity (b) and vocalizations total (c). Infants with ASD compared to TD infants show a reduced rate/min of motor activity at T1 and a reduced rate/min of vocalizations at T2. (b), (c) Means and standard deviations (error bars). **P ≤ 0.01; *P ≤ 0.05.

Moreover, the analysis of events suggests several qualitative differences in the behaviors used by the infants in order to involve or to respond to the caregiver. First, the early significantly reduced amount of activating behaviors (total and with motor activity) and total motor behaviors in infants with ASD highlights the lack of motor activity used by these infants both to engage or to respond to caregivers' bids (Figure 2). The reduced motor activity at T1 confirms other studies on home videos. Adrien et al. [26] observed higher levels of hypotonia and the presence of unusual postures (see also [27]) in infants with ASD. Phagava et al. [28] described a very early pattern of poor motor repertoire in infants with ASD. Also retrospective interviews with parents of children with ASD support hypotonia and reduced motor activity as early signs of ASD, sometimes years before diagnosis is performed [29]. Some studies on siblings have described decreased activity at six months of life as a characteristic of infants who are developing an ASD [22, 30]. These data on young children are further supported by the meta-analysis carried out by Fournier et al. [31] on older children. We hypothesize that differences in motor activity during the first year of life, and especially during the first 6 months, may be a key component in the understanding of the different quality of reciprocity. Further research could explore to what extent this early lack of motor activity might jeopardize the child's ability to start and to respond to an interactive exchange, making the infant-parent interaction more difficult and compromising the development of reciprocity.

In the second six months, the same activating and motor behaviors significantly increase in infants with ASD as well as in TD infants, although they remain at a slightly lower level in ASD, and attuned motor activity significantly increases only in TD infants. This longitudinal data might suggest that, even if there is an improvement of motor activity in ASD, these children make less use of it to be attuned to others. We could hypothesize that the pattern, composed of an early lower level of motor activity and a subsequent nonincrease in attuned motor activity, could represent the expression or the substrate of ASD difficulties in reciprocity, which in TD infants relies on the significant increase of attuned behaviors—with motor activity.

While in the first six months we only found differences in items referring to motor activity, during the second six months of life, lower rates in the amount of vocalizations—total significantly distinguished ASD from TD (Figure 2). This difference was due to both the significant decrease in vocalizations, in particular of the responsiveness type, in the ASD group and to their significant increase in the TD group. The absence in the ASD group of a significant increase in vocalizations to respond is in agreement with the general absence of an increase in the duration of the state responsiveness that we have described above.

This result, showing an atypical pattern of vocalization development during the first year of life, is comparable to that of a prospective study by Ozonoff et al. [32] on siblings who later developed an ASD, where it was found that at the age of six months there were no significant differences in the frequency of either vocalizations or smiles compared to TD infants (indeed the values of children with ASD were slightly higher, as in our sample), while in the second six months a reduction in frequency occurred that resulted in significant differences by the age of twelve months. Similarly, Goldberg et al. [33] have reported that very often parents refer, with a good level of accuracy, their child's regression in the domain of early vocalizations. These different results for early vocalizations could also be interesting in the light of Grossman's study [34] which provided evidence of a voice-sensitive region in the brain of 7-month-old, but not 4-month-old, infants. Other studies as well as ours go in the same direction as far as typical development is concerned, in fact in TD infants an important increase in vocalizations was found between the first and the second six months of life whereas the reduction in the total amount of vocalizations in the ASD group could signify a failure in this developmental step. The reduced use of vocalizations to respond could suggest that, in infants with ASD, the original vocalizations do not develop into a vocal communication able to respond, engage in dialogue, evolving ultimately into structured language. Congruently with the four to seven month period described as critical for the development of both language [35] and cerebral voice processing [34], the atypical pattern of vocalization found in the ASD group highlights the period between the first and the second six months as critical for the development of autism.

**Figure 3:**
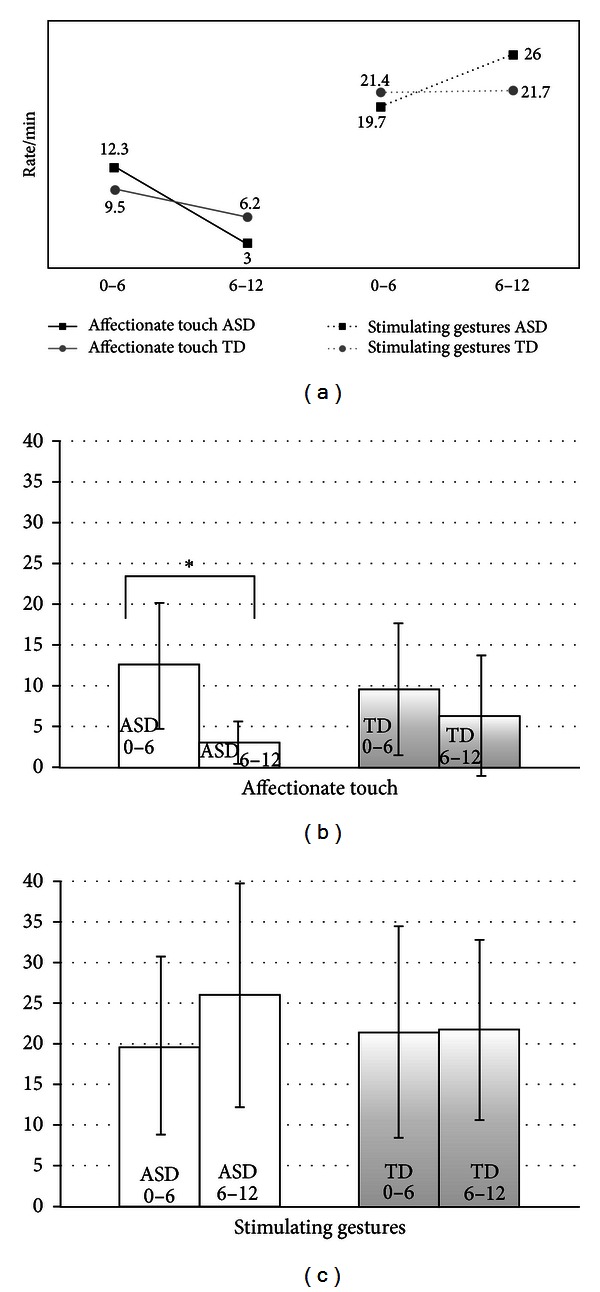
Trajectories of caregiver's affectionate touch verses caregiver's stimulating gestures. (a) Trajectories (from T1 to T2) of affectionate touch (b) and stimulating gestures (c). In the transition from T1 to T2, caregivers of infants with ASD compared to caregivers of TD infants show a significant reduction of affectionate touch, whereas the rate/min of stimulating gestures increases. (b), (c) Means and standard deviations (error bars). **P ≤ 0.01; *P ≤ 0.05.

As for caregiver behaviors, the most significant result was the reduction, between T1 and T2, of affectionate touch in the ASD group. Affectionate touch refers to caregivers touching the infant in an affectionate way (i.e., caressing or kissing him/her). A natural and progressive decrease in caregivers' affectionate touch during the first year of life of typical children has been described by Ferber et al. [36]. We also found a decrease in this behavior in our TD group; however, the decrease is less rapid and does not reach a significant level as it does for caregivers of infants with ASD. The different trajectory of affectionate touch from T1 to T2 (Figure 3) is in agreement with the significant decrease from T1 to T2 in the duration of the state involvement. We could hypothesize that the decrease in involvement and affectionate touch is probably related to the convergent action of the atypical motor and vocal development during the first year of life of infants with ASD. One hypothesis could be that caregivers decrease their use of touch to involve their infants as a result of the reduced responsiveness and attuned behaviors described in children with ASD. Another hypothesis to take into account concerns the caregiver's inherent social skills which are likely to be different across subjects and especially across groups, as ASD is a dimensional trait that runs in families. However, more powerful analyses should be used to validate these considerations. In Figure 3, it is also possible to observe the opposite trajectory of the caregiver's stimulating gestures (caregiver's gesticulating, tickling, making faces, or presenting objects to the infant). In fact, caregivers of infants with ASD present an increase in stimulating gestures to involve their infants, that is, not present in the TD group. Two of our previous studies on home movies pointed to the increase in parental solicitations (both “regulating up” and “touching”) as an early marker for ASD [11, 14]. Moreover, a recent paper on parent-infant interaction in infant siblings at risk for autism has shown that caregivers' interactive behaviors are more directive [10]. The current study adds another aspect to these previous findings on the way parents of infants with ASD adapt to their less responsive child, which might appear contradictory. On the one hand, parents of infants who are developing an ASD might consider their children's behavior to be a reflection of his/her temperamental attitude and adapt their behavior by reducing affectionate touch; on the other hand, they might recognize that something is going wrong in their too passive and unresponsive infant and congruently increase their stimulating attitude. Nevertheless, because the infant's behaviors are reduced in terms of quality but not in the duration of involvement and responsiveness, parents might notice only some slight differences and slowly grow in their awareness that their child needs further solicitation. In fact, one methodological aspect regarding the study conducted by Saint-Georges et al. [14] is crucial: the use of computational methods permitted the selection of only successful interactive patterns (meaning a caregiver solicitation followed by an infant response). It is in this specific context that caregiver touching and regulating up is significantly increased in parents of infants with ASD compared to parents of TD infants.

## 5. Limitations

The first and most relevant limitation is represented by the small sample size, as the Type I error cannot be ruled out and the results may not survive multiple comparison tests. Moreover, we have described different patterns in the two groups by carrying out separate analyses of differences between and within groups through Student's *t*-test, but it was not possible to perform a formal statistical test of the interaction between these differences. Given the small sample size and the limited power of the statistical analysis, results should be considered a testable hypothesis for future adequately powered studies.

A second limitation is the quality of the videotapes recorded by parents in a *naive *context, which varies across subjects and reduces the comparability of the sequences in terms of opportunity for reciprocal exchanges. To reduce the bias of the wide variability of video sequences, we chose situations of interaction between infant and caregiver as similar to each other as possible, selecting the video material specifically on the basis of the scenes which were filmed.

Finally, a further limitation is the absence of a control group consisting of infants with non-ASD neurodevelopmental disorders (e.g., intellectual disability). In our study, ASD and TD groups were not matched based on IQ, thus we cannot exclude the possibility that the differences are in part due to this factor. Therefore, we are aware that this study lacks evidence for the specificity of the abnormalities found in infants with ASD.

## 6. Conclusions

To summarize, the present study suggests that infants with early onset autism spectrum disorders may display both motor and vocal impairments. We hypothesize that a nonsynchronic motor-vocal pattern may interfere in different ways with the development of reciprocity in the primary relationship between infants later diagnosed with ASD and their caregivers. Differences in motor activity patterns are evident by the end of the first six months of life, and we suggest that they may have a role in the lack of initiative and in difficulties in having an active role in purposeful interactions as well as in reciprocal responses. On the other hand, a reduction of vocalizations across time and in comparison with TD infants may be seen as a red flag of atypical social development.

Given the limitations, these findings should be regarded as very preliminary, encouraging further research in this direction with a dual purpose: to evaluate if the differences we have highlighted withstand more powerful statistical analysis by increasing the sample size and to determine the specificity of the results by introducing a control group of infants with non-ASD developmental disorders. Moreover, future research addressing the topic of infants' motor-vocal development might benefit from a prospective approach which would enable the use of standardized observations of infants at risk.

If this nonsynchronic motor-vocal pattern observed in infants later diagnosed with ASD were to be confirmed by future studies, its early identification might provide useful information for determining infants at risk for ASD and starting a timely intervention.

## Figures and Tables

**Table 1:** Caregiver-infant rating scale.

Infant behaviors		

States^(1)^	Involvement	A time interval in which the child tries to attract the caregiver's attention and to retain it. Involvement starts when the child solicits an interaction and stops when the child shifts his/her attention toward an object and abandons his/her active role in the interaction, possibly moving onto a responsiveness state. * For example, the child starts to look and vocalize toward the caregiver while he/she is talking to someone else in the room and not looking at the child. *
	Responsiveness	A time interval in which the child appears disposed to respond appropriately to the caregiver's attempts to attract his/her attention. Responsiveness starts when the child accepts the caregiver's invitation to participate in the interaction and stops when he/she is no longer interested in the other or when he/she shifts his/her gaze and directs his/her attention away or takes an active role, starting an involvement state. * For example, the child is looking at an object and the caregiver calls his/her name, looks at and smiles at him/her in order to obtain his/her gaze and attention. The child shifts his/her gaze towards the caregiver and starts to vocalize towards him/her. *
Events^(2)^	Vocalization (a) of involvement (b) of responsiveness	The child vocalizes and looks toward the caregiver. Only vocalizations directed to the caregiver are considered. Vocalizations directed toward objects or toward someone different from the caregiver (undirected) are not coded. Vocalizations are distinguished according to their function in the interaction. (a) Vocalizations are used to start an interaction or to attract the caregiver's attention (b) Vocalizations are used to respond to the caregiver's solicitations.
	Activating behavior (a) with gaze (b) with motor activity	The child provokes an interaction in order to involve the caregiver. For this purpose he/she can (a) orient their gaze toward the caregiver, (b) perform motor activity toward the caregiver (i.e., moving limbs, touching, grabbing, reaching out, and moving toward). Motor activity is considered as an activating behavior only if it is used with the purpose of attracting the caregiver's attention. Spontaneous movements are not coded.
	Attuned behavior (a) with gaze (b) with motor activity	The child responds to the caregiver's attempts to attract his/her attention, For this purpose he/she can (a) orient their gaze toward the caregiver when the latter solicits his/her attention, (b) perform motor activity directed towards the caregiver (i.e., adapting posture to caregiver's body, moving limbs, reaching out, etc.) Motor activity is considered as an attuned behavior only if it is used with the purpose of responding to the caregiver's stimulations. Spontaneous movements are not coded.
	Smile (a) Coordinated (b) Uncoordinated	The infant smiles in different ways. The smile is distinguished according to the direction of the gaze. (a) The infant's smile is coordinated with the direction of his/her gaze towards the caregiver. (b) The infant's smile is not coordinated with his/her gaze towards the caregiver.

Caregiver behaviors		

States^(1)^	Involvement	A time interval in which the caregiver tries to attract the infant's attention and to retain it. Involvement starts when the caregiver solicits the infant to participate in an interaction and stops when he/she abandons his/her active role (i.e., the caregiver directs his/her attention
		toward an object or abandons his/her active role in the interaction changing to a responsiveness state). * For example, while the child is looking at an object, the caregiver calls his/her name in order to obtain his/her gaze and his/her attention, or he/she looks or smiles at him/her. *
	Responsiveness	A time interval in which the caregiver responds appropriately to the child's attempts to attract his/her attention and to retain it. Responsiveness starts when the caregiver responds to the infant's invitation by participating in an interaction and stops when he/she is no longer interested in the interaction or when he/she changes his/her State (i.e., when he/she changes to an involvement state). * For example, the caregiver is talking with someone else in the room and he/she is not looking at the child. The child starts to look at the caregiver and vocalizes toward him/her in order to obtain his/her attention, and the caregiver responds by looking at the child and smiling or vocalizing toward him/her. *
Events^(2)^	Vocalization (a) of involvement (b) of responsiveness	The caregiver vocalizes towards the infant, looking at him/her. Only vocalizations directed to the child are considered. Vocalizations directed toward objects or toward someone different from the child (undirected) are not coded. Vocalizations are distinguished according to their function in the interaction. (a) Vocalizations are used to start an interaction or to attract the child's attention. (b) Vocalizations are used to respond to the child's solicitations.
	Name prompt	The caregiver calls the infant by name.
	Affectionate touch (a) of involvement (b) of responsiveness	The caregiver touches the infant in an affectionate way, for example, caressing or kissing him/her. Affectionate touch is also distinguished according to its function in the interaction. (a) Touch is used to start an interaction or to attract the child's attention. (b) Touch is used to respond to the child's solicitations.
	Stimulating gesture (a) of involvement (b) of responsiveness	The caregiver gesticulates, tickles, makes faces, or presents the infant with objects. Stimulating gestures are distinguished according to their function in the interaction. (a) Gestures are used to start an interaction or to attract the child's attention. (b) Gestures are used to respond to the child's solicitations.

^(1)^Duration and frequency are considered.

^(2)^Frequency is considered.

**Table 2:** Infant's behaviors: means and standard deviations for ASD and TD. *t*-test between groups at T1 and T2 and within groups in ASD and TD.

Infant's behaviors	T1^(1)^		T2^(2)^		*t*-test between groups		*t*-test within groups	
	*M* (SD)		*M* (SD)		*P* value^(3)^		*P* value^(3)^	
	ASD	TD	ASD	TD	T1^(1)^	T2^(2)^	ASD	TD
States^(4)^								

Involvement								
% duration	3,01 (5,4)	8,3 (10,7)	5,8 (9,9)	9,1 (5,7)	0,18	0,39	0,41	0,85
rate/min	0,3 (0,6)	0,6 (0,7)	0,5 (0,9)	0,9 (0,5)	0,32	0,25	0,48	0,19
Responsiveness								
% duration	54,7 (26)	46,3 (23)	53,3 (30,1)	71,6 (17,1)	0,47	1,13	0,86	0,03*
rate/min	2,2 (1,7)	2,4 (1,6)	1,6 (0,7)	2 (0,8)	0,77	0,36	0,38	0,39

Events^(5)^								

Activating behaviors								
Total	0,8 (1,1)	2,8 (2,8)	5,4 (5,1)	5 (3,1)	0,04*	0,81	0,02*	0,11
Gaze	0,3 (0,4)	1,1 (1,3)	1,6 (2,1)	1,1 (1,2)	0,08	0,53	0,07	0,95
Motor	0,5 (0,7)	1,8 (1,9)	3,8 (3,5)	3,9 (2,1)	0,07	0,99	0,02*	0,02*
Attuned behaviors								
Total	14,6 (7,8)	18,5 (6,5)	17,4 (10,6)	23,6 (11,1)	0,25	0,06	0,21	0,16
Gaze	9,2 (4,6)	10,4 (5,1)	6,3 (5,7)	7,7 (6,3)	0,60	0,55	0,57	0,65
Motor	5,3 (4,1)	8,1 (3,3)	7,1 (6,7)	14,5 (9,1)	0,13	0,23	0,24	0,05*
Vocalizations								
Total	15,6 (11,6)	14,3 (9,1)	10,1 (10,2)	26,5 (11,1)	0,80	0,004**	0,03*	0,03*
Involvement	2,2 (4)	5,8 (8)	3,1 (4,3)	6,9 (3,8)	0,22	0,06	0,59	0,68
Responsiveness	13,3 (11)	8,5 (6,3)	7 (8,1)	18,3 (12,5)	0,26	0,03*	0,07	0,02*
Smile								
Total	7,6 (8,2)	5,8 (4,4)	4,3 (4,1)	7,4 (5,2)	0,56	0,17	0,19	0,37
Coordinated	5,6 (7,2)	4,6 (3,1)	2,8 (2,9)	3,1 (2,2)	0,69	0,80	0,21	0,20
Uncoordinated	1,1 (1,2)	0,7 (1,3)	0,8 (1,1)	1,6 (2)	0,56	0,24	0,51	0,29

Total Gaze^(6)^	9,5 (4,7)	11,5 (5,5)	11,9 (6,8)	10,2 (7,2)	0,40	0,61	0,34	0,69
Total Motor^(7)^	5,9 (4,1)	9,8 (3,9)	10,9 (6,6)	18,4 (9,8)	0,04*	0,07	0,02*	0,02*

^(1)^0–6 months period.

^(2)^6–12 months period.

^(3)∗∗^ *P* ≤ 0.01; *P ≤ 0.05.

^(4)^State: the time interval in which the behavior is observed. Duration and frequency are considered.

^(5)^Events: single behaviors coded each time they are observed. Frequency is considered.

^(6,7)^These two additional items were created merging motor and gaze behaviors allocated in the activating and attuned behaviors.

**Table 3:** Caregiver's behaviors: means and standard deviations for ASD and TD. *t*-test between groups at T1 and T2 and within groups in ASD and TD.

Caregiver's behaviors	T1^(1)^		T2^(2)^		*t*-test between groups		*t*-test within groups	
	*M* (SD)		*M* (SD)		*P* value^(3)^		*P* value^(3)^	
	ASD	TD	ASD	TD	ASD	TD	ASD	TD
States^(4)^								

Involvement								
% duration	87 (14,1)	76,9 (15,5)	66,6 (24)	82,4 (9,7)	0,15	0,08	0,04*	0,43
rate/min	2,4 (1,1)	7,9 (11,2)	3,5 (7,3)	5,6 (6,5)	0,31	0,52	0,95	0,64
Responsiveness								
% duration	3,7 (1,1)	3 (1,8)	1,9 (0,8)	2,2 (1)	0,40	0,56	0,41	0,23
rate/min	0,3 (0,4)	0,6 (0,7)	0,3 (0,7)	0,4 (0,4)	0,32	0,79	0,87	0,57

Events^(5)^								

Affectionate touch								
Total	12,3 (7,7)	9,5 (8,1)	3 (2,6)	6,2 (7,4)	0,45	0,21	0,00**	0,53
Involvement	12,2 (7,6)	8,5 (7,4)	2,9 (2,4)	6,1 (7,3)	0,29	0,20	0,00**	0,09
Responsiveness	0,04 (0,1)	1,02 (1,4)	0,11 (0,2)	0,1 (0,2)	0,05*	0,82	0,51	0,42
Vocalizations								
Total	63,3 (29,9)	55,5 (24,1)	47,5 (34,5)	66,5 (25,2)	0,54	0,69	0,17	0,54
Involvement	59,2 (29,8)	49,1 (24,5)	44,4 (36,2)	56,5 (22,5)	0,44	0,40	0,22	0,97
Responsiveness	4,1 (4,3)	6,4 (8,2)	3,1 (3,8)	6,4 (8,4)	0,44	0,27	0,60	0,34
Stimulating gestures								
Total	19,7 (10,9)	21,4 (13,1)	26 (13,7)	21,7 (11,1)	0,77	0,46	0,36	0,84
Involvement	19,6 (11)	20,8 (13,2	24,8 (14,1)	19,7 (10,9)	0,82	0,39	0,44	0,12
Responsiveness	0,1 (0,3)	0,5 (0,7)	1,2 (1,4)	1,9 (2,6)	0,16	0,41	0,07	0,95
Name prompt	2,7 (3,6)	2,4 (1,4)	3,1 (2,4)	2,6 (1,5)	0,82	0,55	0,72	0,83

^(1)^0–6 months period.

^(2)^6–12 months period.

^(3)∗∗^ *P* ≤ 0.01; *P ≤ 0.05.

^(4)^State: the time interval in which the behavior is observed. Duration and frequency are considered.

^(5)^Events: single behaviors coded each time they are observed. Frequency is considered.
